# Mind wandering simultaneously prolongs reactions and promotes creative incubation

**DOI:** 10.1038/s41598-017-10616-3

**Published:** 2017-08-31

**Authors:** Marcin Leszczynski, Leila Chaieb, Thomas P. Reber, Marlene Derner, Nikolai Axmacher, Juergen Fell

**Affiliations:** 1 Department of Neurological Surgery, Columbia University Medical Center, New York, USA; 2 Translational Cognitive Neuroscience Program, Nathan Kline Institute, Orangeburg, New York, USA; 3 Department of Epileptology, University of Bonn, Bonn, Germany; 4 Department of Neuropsychology, Institute of Cognitive Neuroscience, Ruhr University Bochum, Bochum, Germany

## Abstract

Mind wandering (MW) refers to the disengagement of attention from the external environment and the generation of thoughts unrelated to the task at hand. It is a ubiquitous cognitive process resulting in lapses of attention. MW imposes a negative impact on attention-based task performance, but also has been associated with enhanced creativity and future planning. In three experiments we show that MW relates simultaneously to both behavioral costs but also benefits. Behavioral costs were measured by prolonged reaction times (RT) in sustained attention to response tasks (SART), whereas the benefits were observed as improved performance in the creative problem solving and daily routine planning tasks performed after the SART. Additionally, we found an increased dispersion of RTs during MW suggesting that attention during these times underwent dynamical changes compared to states when participants were fully focused on the task. Our results support a model in which MW deteriorates performance in the task at hand and is related to dynamical changes in attention. At the same time it is also able to improve human capacity for complex operations.

## Introduction

It has been known for a long time that the minds of humans wander a lot^1, 2^, meaning that they have thoughts divagating from the current task or situation. However, it is only recently that this cognitive phenomenon has been properly quantified revealing that mind wandering occupies up to half of our waking time^3^. Mind wandering comes at high costs: it causes errors and delays in task performance and has a negative impact on mood^3, 4^. Ultimately, mind wandering can lead to injuries or death, for instance, when occurring during driving a vehicle (e.g. ref. 5). The ubiquity of mind wandering suggests that this cognitive activity should also have major benefits. Otherwise, engaging in it would be very disadvantageous and would result in a high evolutionary pressure to eliminate it. Indeed, indirect evidence supports a relationship between individual differences in traits related to mind wandering and creativity, as well as the planning of future activities^4^. Baird and colleagues^6^ have shown that more solutions for a creative problem (unusual uses task) could be generated after a break filled with an undemanding task, which is known to stimulate mind wandering, compared to breaks filled with a demanding task, pure rest or no break. Zedelius and Schooler^7^ have reported that an increased individual tendency to mind wander was positively related to solving creative problems with a sudden insight (“aha”) experience rather than gradually arriving at a solution using an analytic strategy. Ruby and colleagues linked mind wandering to better social problem solving^8^ and suggested that these two processes both depend on sensory disengagement. Future oriented mind wandering was also found to correlate with the development of more concrete personal goals^9^.

Although these findings suggest that mind wandering might result in both positive and negative outcomes, no previous study has investigated whether these distinct effects are occurring at the same time. Yet, studying the concurrent presence of opposing effects is necessary to understanding the twofold nature of mind wandering. Here we examined the simultaneous occurrence and interdependence of these opposing effects. For this purpose, sustained attention to response tasks (SART; ref. 10) were nested into the incubation periods of creative problem solving and daily routine planning tasks. Mind wandering during the SART tasks was quantified by an experience sampling procedure. This design allowed us to study the impact of mind wandering on reaction times, as well as the influence of the number of mind wandering instances on performance in the creative problem solving (exp. 1 and 2) and daily planning tasks (exp. 3). Moreover, we investigated if the effect of mind wandering on creative problem solving depended on whether unsolved items of the problem solving task are incorporated into the SART task (exp. 1) or not (exp. 2), i.e. whether mind wandering on the creative problems can be actively stimulated or not. One prominent hypothesis suggests that mind wandering involves sensory disengagement and attention relocation from current sensory information to episodic and semantic memory representations^11, 12^. We reasoned that actively stimulating the semantic representations of unsolved compound remote associates (CRA) items during the SART might facilitate creative incubation and thereby foster a positive influence of mind wandering on post-SART CRA performance (see Methods).

Furthermore, several frameworks propose that mind wandering is a heterogeneous cognitive state. It has been suggested to consist of a collection of components^11, 12^ or a collection of thoughts through which the mind wanders^13^. These different components might in turn be reflected by different levels of disengagement from the current task (and different demands on cognitive control). One possible way to measure such dynamical changes is through intraindividual variation in reaction times to an ongoing task, e.g. the SART. In fact increased response variability has been related to attentional lapses and changes in executive control^14, 15^. Therefore, if mind wandering consists of a collection of components with varying demands onto executive control, one may expect an increased dispersion of reaction times during off-task periods. However, it has not yet been investigated whether the dispersion of reaction times during mind wandering is increased compared to mind wandering-free intervals.

To summarize, we investigated the following hypotheses in three independent experiments: (a) Mind wandering causes prolonged reaction times in SART tasks; (b) at the same time, the numbers of mind wandering instances during SART tasks are positively correlated with performance in the creative problem solving and daily planning tasks; (c) the impact of mind wandering on creative problem solving is larger when unsolved items of the compound remote associates task are used as non-target stimuli in the SART task; (d) the dispersion of reaction times is greater for off-task intervals (mind wandering) than for on-task intervals (no mind wandering). In the context of our study, we use the terms mind wandering and off-task interchangeably to indicate a state, where attention has been withdrawn from the SART task.

## Results

**Figure 1:**
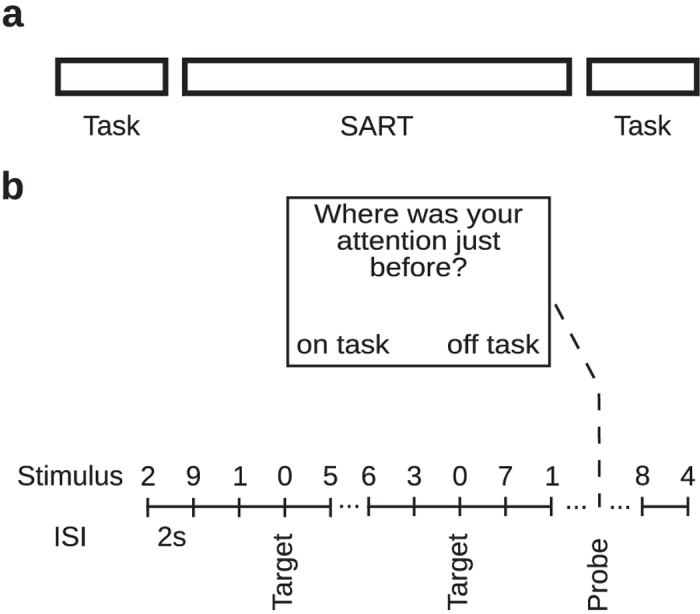
Experimental task. (**a**) Overall design for experiments 1–3. Sustained attention to response (SART) tasks were embedded into the incubation periods of a creative problem solving task (experiment 1 and 2) and a daily planning task (experiment 3). (**b**) An example of a stimulus sequence in the SART. Digits 1–9 were used as non-targets (i.e. required button presses), while 0 was a target which required participants to withhold their reaction. The digits were presented in a random order with 11% probability of targets throughout the experiment. Participants were instructed to respond as quickly and accurately as possible. The experience probes were presented at random time points during the SART with each inter-probe interval selected from the range of 40–60 sec.

To test these hypotheses we embedded a SART task into the incubation periods of creative problem solving and daily planning tasks (Fig. 1a). During the SART task we applied an experience sampling procedure which enabled us to measure the number of mind wandering instances and directly relate it to the number of creative insights (Fig. 1b).

**Figure 2:**
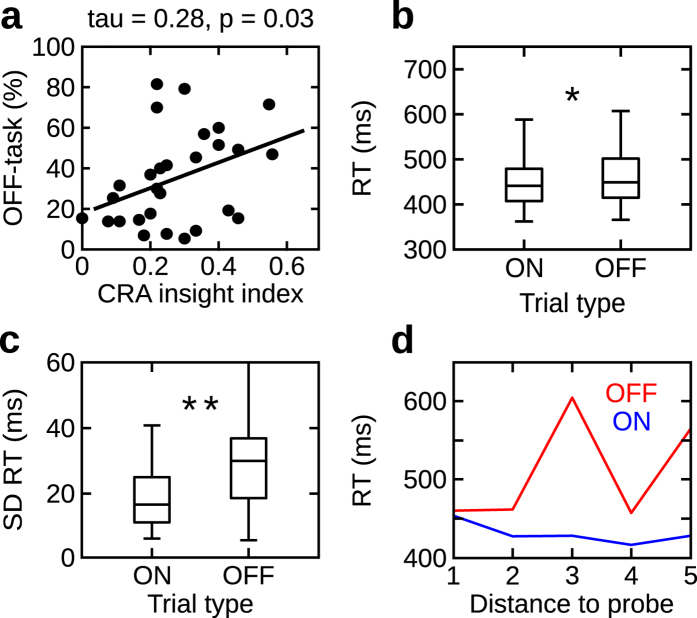
Results experiment 1. (**a**) Correlation between the number of mind wandering instances and creative insight index (N = 28). (**b**) Box plot of reaction times separately for on- and off-task states (no mind wandering and mind wandering, respectively). The central line and edges reflect median and 25^th^, 75^th^ percentiles, respectively. The whiskers extend to the most extreme data points. (**c**) Box plot of standard deviation of reaction times separately for on- and off-task (no mind wandering and mind wandering, respectively) intervals. The central line and edges reflect median and 25^th^, 75^th^ percentiles, respectively. The whiskers extend to the most extreme data points. (**d**) Example of single participant data illustrating the effect of increased reaction time dispersion during mind wandering. Reaction times for five consecutive responses preceding the experience sampling probe plotted separately for no mind wandering (on-task; blue) and mind wandering (off-task; red) responses to the experience sampling probe. *Indicates p < 0.05; **indicates p < 0.01.

In experiment 1, participants responded on average 34% of the time as being off-task (exp. 2: 47%; exp. 3: 48%). We found that the amount of mind wandering positively correlated with the number of creative insights (Kendall’s tau = 0.28, p = 0.03: N = 28; Fig. 2a). This result indicates that participants who were more prone to mind wander were able to solve more word triplets in the post-SART compound remote association task, which in turn, suggests that mind wandering improves the capacity for creative problem solving. Critically, no correlation was observed between the amount of mind wandering and the number of creative solutions provided in the pre-SART session (Kendall’s tau = 0.12; p = 0.36, N = 28; Fig. 5a). This is important to note, as it suggests that increased mind wandering during the SART directly relates to the improved creative problem solving performance in the post-SART session. This also excludes the possibility that we measured a general trait of subjects who mind wander more to be more creative. This would be the case if the correlation between the amount of mind wandering and the number of correct creative solutions would be also observed in the pre-SART session. It should be noted that we do not imply that there exists no correlation between the amount of mind wandering and pre-SART performance. The present finding shows that in the current design, this relation is at least substantially weaker than the correlation between the amount of mind wandering and post-SART performance. As expected mind wandering had a detrimental influence on performance in the SART task. This was measured by increased reaction times during mind wandering compared to task-focused intervals. Using a Wilcoxon signed rank test (see Methods) we observed increased reaction times during off- as compared to on-task intervals (median off-task vs. on-task: 448 ms vs. 439 ms z = 2.43, p = 0.01; r = 0.32; N = 28; Fig. 2b). Kruskal-Wallis test showed no difference in reaction times as a function of time before the on- (χ^2^(4) = 0.21, p = 0.99; N = 28) and off-probe (χ^2^(4) = 0.92, p = 0.92; N = 28). Furthermore, we found that reaction times during off states (i.e., over the five consecutive responses preceding the experience sampling probe) exhibited a larger dispersion, in terms of standard deviation, than reaction times during on states. This effect was evident when comparing standard deviations calculated across these five responses preceding the off and on responses (median 29 ms vs. 17 ms; Wilcoxon signed rank test: z = 3.14, p = 0.001, r = 0.41; N = 28; Fig. 2c,d). We also calculated accuracy scores for on and off intervals. This analysis further supports our observations derived from reaction times. Accuracies were very high overall (i.e. >95%) which was to be expected as the task was designed to be easy to perform. Importantly, a Wilcoxon signed rank test showed that participants were more accurate during on-task compared to off-task intervals (99% vs. 96%; z = 3.45, p < 0.001; r = 0.46; N = 28). Similarly to our reaction time results, the variance of accuracy was increased during off- compared to on-task intervals. This was revealed by a significant Wilcoxon signed rank test (off- vs. on-task: 3.2% vs. 1.1%; z = 3.17, p = 0.001; r = 0.42; N = 28). Altogether, this outcome supports a dynamic framework of mind wandering and suggests that off-task periods are heterogeneous cognitive states with increased attentional fluctuations.

**Figure 3:**
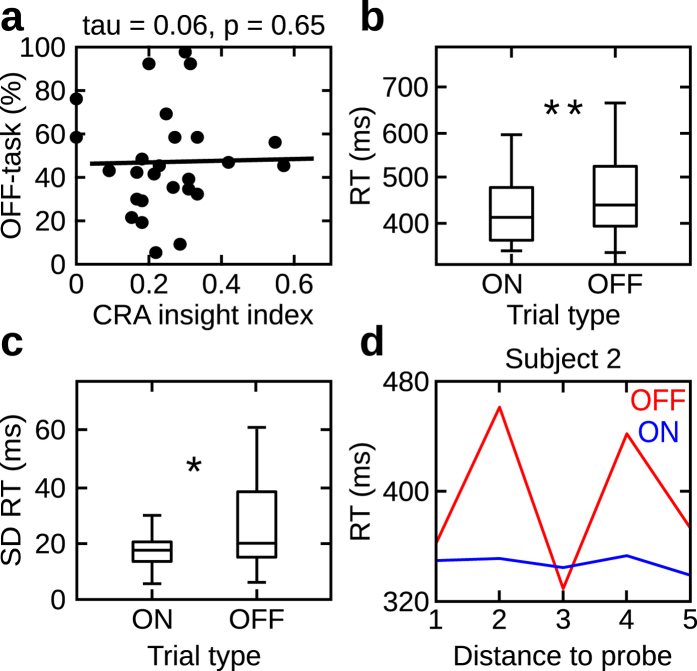
Results experiment 2. Same as Fig. 2 showing results in experiment 2 (N = 26). *Indicates p < 0.05; **indicates p < 0.01.

The SART task in experiment 1 used stimuli contingent upon word items used in the creative problem solving test (outlined in the Methods section). In experiment 2 we investigated whether the beneficial effect of mind wandering could be observed when both tasks are independent of one another. To this end, a naive group of participants performed the same creative task (CRA). However, during the SART, participants were instructed to withhold a button press when a target digit (number 0) was presented amongst the continued presentation of non target digits (numbers 1–9). Interestingly, the correlation between the amount of mind wandering and the number of creative insights was not significant (Kendall’s tau = 0.06, p = 0.65, N = 26; Fig. 3a). This suggests that actively stimulating mind wandering has a particularly beneficial influence on creative incubation. Importantly, we again observed no correlation between the amount of mind wandering and the number of creative solutions provided in the pre-SART session (Kendall’s tau = 0.08; p = 0.59, N = 26; Fig. 5b). In experiment 2, we also observed that mind wandering impaired performance in the SART. Wilcoxon signed rank test showed that reactions times were increased during off as compared to on states (median off-task vs. on-task: 437 ms vs. 410 ms; z = 3.84, p < 0.001, r = 0.53, N = 26; Fig. 3b). No difference in reaction times as a function of time before the on- (Kruskal-Wallis test: χ^2^(4) = 0.46, p = 0.97; N = 26) and off-probe (Kruskal-Wallis test: χ^2^(4) = 1.21, p = 0.87; N = 26) was found. The effect of increased standard deviation of reaction times during off states compared to on states was significant (median: 19 ms vs. 17 ms; Wilcoxon signed rank test: z = 1.99, p = 0.04; r = 0.27; N = 26; Fig. 3c,d). Accuracy further supported the reaction time results showing an increased number of correct responses during on- as compared to off-task intervals (median 99% vs. 97%; z = 3.44, p < 0.001, r = 0.48, N = 26). Finally, the variance of accuracy was also increased during off- compared to on-task intervals (1.7% vs. 0.8%; z = 3.13, p = 0.001, r = 0.43, N = 26). These results show that the twofold simultaneous influence of mind wandering on behavior is only observed when creative insights are actively stimulated during SART (exp. 1). In particular, a significant effect of mind wandering on creative problem solving was found in experiment 1 (i.e. creative insights were actively stimulated during SART), but not in experiment 2 (i.e. creative insights were not actively stimulated during SART) with all other factors kept constant. These results support hypothesis c) that incorporating CRA items into the SART task triggers more creative insights.

**Figure 4:**
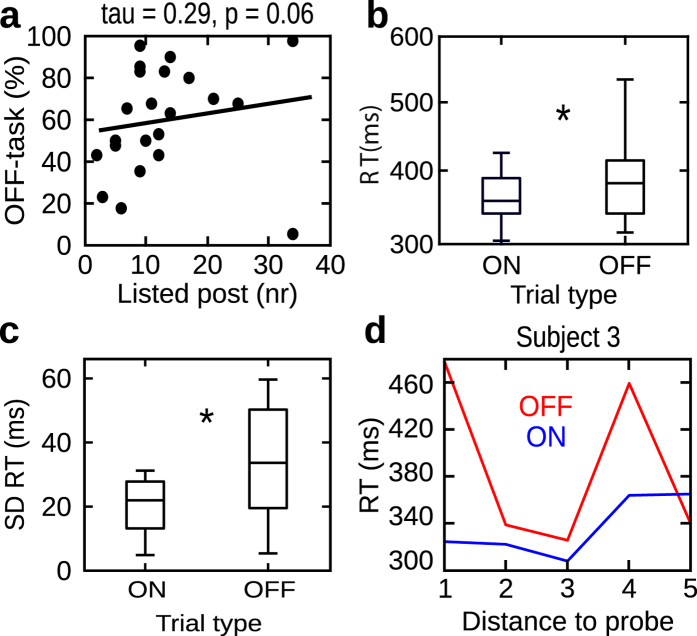
Results experiment 3. Same as Figs 2 and 3 showing results in experiment 3 (N = 22). *Indicates p < 0.05.

**Figure 5:**
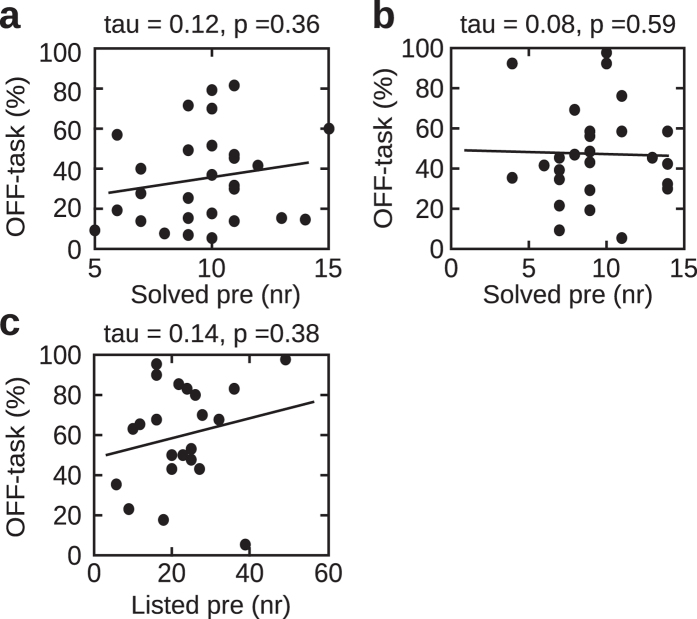
Correlations between pre-SART performance and the amount of mind wandering. Correlation between the amount of mind wandering and the number of solved CRA items before the SART session in experiment 1 (**a**), 2 (**b**) and between the amount of mind wandering and the number of items listed in the DPT﻿ before the SART session in experiment 3 (**c**).

In experiment 3 we explored whether the benefits of mind wandering observed in experiment 1 also generalize to another form of cognitive activity, namely future planning of daily routines. In contrast to the problem solving tasks used in experiments 1 and 2, this task did not require finding a correct solution, but asked participants to outline planned activities over the course of the next seven days. We hypothesized that participants who mind wandered more often would be able to list more routine activities, after they had performed the SART task. Indeed, we found a positive trend between the number of mind wandering instances and the number of items added to the daily routine list (Kendall’s tau = 0.29, p = 0.06; N = 22; Fig. 4a). The correlation between the amount of mind wandering and the number of items listed in the pre-SART session was not significant (Kendall’s tau = 0.14; p = 0.38; N = 22; Fig. 5). This again supports our claim that it is the increased mind wandering during the SART, which directly relates to the improved performance in the post-SART session. As in the case of experiments 1 and 2, we observed that mind wandering impaired performance during the SART. Using a Wilcoxon signed rank test we observed increased reaction times during off- compared to on-task intervals (median off-task vs. on-task: 378 ms vs. 352 ms; z = 2.09, p = 0.04, r = 0.30, N = 22; Fig. 4b). Again, no difference in reaction times as a function of time before the on- (Kruskal-Wallis test: χ^2^(4) = 1.13, p = 0.88; N = 22) and off-response (Kruskal-Wallis test: χ^2^(4) = 1.46, p = 0.83; N = 22) was found. The effect of increased standard deviations of RTs during off states compared to on states was also significant (33 ms vs. 21 ms; z = 2.12, p = 0.03; N = 22; Fig. 4c,d). Accuracy was overall showing a ceiling effect in this experiment with no difference between on- and off-task intervals (median 100% vs. off 98%; z = 0.85, p = 0.39, N = 22). Similarly, the variance of accuracy showed a floor effect (on vs. off: 0% vs. 2.4%; z = 0.96, p = 0.33, N = 22). In experiment 3, we also probed participants for different inner experiences during mind wandering (see Methods). We observed a difference among the frequencies of these types of mind wandering using a Kruskal-Wallis test (χ^2^(2) = 9.30, p = 0.009; median scores were 7.5%, 10% and 20% for inner seeing, feelings and inner speech, respectively). We used a Wilcoxon signed rank test for post-hoc comparisons across pairs of inner experience and found that participants reported to experience inner speech more frequently than inner seeing (z = 2.63; p = 0.008; r = 0.39, N = 22) and feelings (z = 2.06, p = 0.03, r = 0.31, N = 22). No difference was observed between inner seeing and feelings (z = 0.56, p = 0.57, N = 22).

Taken together, the results from experiments 1, 2 and 3 show that mind wandering is detrimental to ongoing task performance in terms of decreased reaction times (confirming hypothesis a). During mind wandering participants not only exhibited slower responses, but also showed a larger dispersion of reaction times compared to non-mind wandering intervals, confirming hypothesis (d). Importantly, we also demonstrate that mind wandering at the same time can promote creative problem solving and future planning, so that insights and solutions are more attainable afterwards (confirming hypothesis b). However, the impact of mind wandering was only observed when items of the problem solving task are incorporated into the SART task (providing support for hypothesis c). Our data show that mind wandering can simultaneously exert detrimental effects on the task at hand, and beneficial effects on performance in another task (e.g. creative problem solving or daily planning). Furthermore, the current results corroborate predictions from a dynamic framework which posits that mind wandering is a heterogeneous cognitive state with increased fluctuations of attention.

## Discussion

Mind wandering is a ubiquitous cognitive state which only recently has received an increased interest among cognitive scientists. Up to now, most mind wandering research has addressed its costs rather than its benefits (for review see ref. 4). Also the prominence of mindfulness-based therapies aiming to lessen mind wandering like mindfulness-based stress reduction^16^ has created the impression that mind wandering primarily has negative effects. The current results provide evidence that mind wandering indeed harms performance in a SART, but simultaneously relates to improved performance in creative tasks. Across three independent experiments we observed that mind wandering has detrimental effects in terms of prolonged reaction times, which is in line with previous findings (e.g. ref. 17). Importantly, at the same time mind wandering directly relates to improved performance in the creative problem solving and future planning tasks. Previous studies have shown that creative insights might be facilitated when people engage in an easy task^6^ or when they adapt a certain strategy^7^ and both conditions are known to be associated with increased mind wandering (see also refs 18 and 19). Moreover, positive effects of mind wandering on anticipation and planning of future events have been reported^20, 21^. Critically, in the current study we embedded SART tasks, which are often used to quantify mind wandering, into creative incubation and daily planning tasks. This allowed us to directly measure the influence of mind wandering on both kinds of tasks (see also ref. 9). Our results support a model in which mind wandering can have a simultaneous twofold influence on human behavior: a negative influence in terms of prolonged reactions and a positive influence related to an increased number of creative insights and more elaborate future planning (see ref. 22). These findings are consistent with prior reports which linked mind-wandering to better social problem solving^8^. Furthermore, Medea *et al*., 2016 observed a correlation indicating that participants developed more concrete personal goals while mind wandering about a future^9^. This effect of goal refinement was further related to increased functional connectivity between the hippocampus and the ventromedial prefrontal cortex. The current findings extend these results showing that mind wandering simultaneously harms performance at hand but also improves creative incubation and daily planning. These findings together with previous results suggest that mind wandering can have both negative, but also positive influence on a broad range of tasks (e.g. creative incubation, daily/future planning, social problem solving).

Although the exact mechanism remains to be discovered these results support a model in which mind wandering is characterized by a cascade of different process components^11, 12, 23^. One important component is the disengagement from external sensory inputs which results in attenuation of sensory processes^24^, and in turn relates to prolonged reaction times and decreased accuracy. This disengagement hypothetically allows the relocation of limited attentional resources to representations independent from the current sensory input, for instance, including episodic and semantic memories^23, 25^. In a broader sense, this disengagement from sensory input may allow the processing of information related to unsolved problems and future goals, which may lead to increased post-SART performance in creative and planning tasks. In this context, we hypothesized that actively stimulating semantic representations of unsolved CRA items during the SART might facilitate creative incubation. Indeed, we found that the correlation between the amount of mind wandering and post-SART problem solving performance was significant only in experiment 1, where we used this approach, but not in experiment 2, where we used a standard SART.

Mind wandering has been hypothesized to be controlled by attentional cycling (i.e. shifts of the attentional focus between the task at hand and task independent thoughts) leading to fluctuations in task performance^26^. Focusing attention on something else than the task at hand is expected to cause delays in reactions as compared to the condition where the full attention is directed to the task. This is indeed what we and others have observed (e.g. refs 4 and 17). However, a recent framework suggests that mind wandering is more complex than just being in a state where attention is directed towards something else than the current task^11–13^. It is a dynamic state during which the mind wanders across several thoughts, feelings and imaginations. This model posits that the state of mind wandering is more heterogeneous compared to the state when attention is fully focused on the task at hand and leads to an interesting hypothesis that reactions might show a higher variability during mind wandering. Indeed, this is what we observed in the current study. One may speculate that this increased dispersion of reaction times, for instance, reflects sequential connected thoughts varying in intensity. This implies that the degree of disengagement of attention from a task is not stable across longer time intervals, but that mind wandering is a process fluctuating on a time scale of several seconds. These fluctuations may have the advantage to minimize the costs of attentional lapses with regard to task performance.

## Methods

### Participants

A total number of 82 adults (50 female) participated in the study (mean age = 23 years, SD+/− = 3.59 years; the number of participants in experiments 1–3 was N = 28, N = 26, N = 28, respectively). Native German speakers were recruited for experiments 1 and 2 as these tasks depended upon semantic skills. The study was approved by the Ethics Committee of the University of Bonn Medical Center. All the methods were performed in accordance with the relevant guidelines and regulations. All participants were informed of the experimental procedure and signed a written consent. Since we intended to compare participants’ responses during times when they were fully focused (i.e., *on the task*) with states of mind wandering (i.e., *off the task*), we included participants who had at least a single response in each of the two conditions. This resulted in a total number of 76 participants across all experiments (N = 28, 26, 22 for experiments 1–3, respectively).

### Tasks and experimental procedure

Each experiment consisted of three parts starting with either the compound remote associates test (CRA; experiments 1 and 2) or a daily planning task (DPT; experiment 3) followed by the sustained attention to response task (SART) and the completion of the CRA and DPT task (Fig. 1a). Each experiment was performed within one session. Participants were informed about all three parts at the beginning of experiment, however the detailed instructions were presented prior to each part.

### The compound remote associates task (CRA)

The CRA is a well-defined task used to measure creative problem solving performance^27^. Participants engaging in the task are shown three target words e.g., “cottage - Swiss - cake” and must solve the problem by finding a shared word associate (in this case ‘cheese’). Each target word triplet is presented once and displayed for maximally 30 seconds, or until the participant responded to the trial. Only one correct solution can be used to form a compound word with each of the three presented words (“cottage cheese, Swiss cheese, cheese cake”). In experiments 1 and 2, a German variant of the CRA was used to measure creative problem solving performance in our participants. A list of 180 CRA items (the target words) and normative data for these items was taken from a standard word item list^28^.

In the current study, we selected 20 CRA items which were solved with an accuracy range between 40 and 50%^28^. The rationale for using items within this range was to keep the task motivating for the participants, while maintaining a level of difficulty that would render some word items unsolvable within the 30 second time frame. Only target word triplets that remained unsolved or were solved incorrectly were presented again to the participants during the post-SART task phase of the CRA. A measure of creative problem solving performance (creative insight index) was computed by dividing the number of CRA items correctly solved in the post-SART task phase through the total number of CRA items presented during the post-SART task. All correlation analyses were performed using Kendall’s tau, a non-parametric rank-correlation test.

### The daily planning task (DPT)

To test whether the beneficial effect of mind wandering detected in experiment 1 generalizes to another type of activity, we developed the Daily Planning task (DPT). The DPT task required participants to outline their daily routines for the next seven days. Participants were instructed to document their activities in a succinct and concise format, e.g. - “walking the dog”. Each item was listed as a bullet point and was not allowed to exceed one line of normalized text. Participants were asked to list as many points as possible within a 5 minutes time frame. Then, they were asked to perform the SART task. After the SART task was completed, they were asked once again to complete their list of routine activities, but listing only new ones that became apparent to them during the SART task. The correlation coefficient between DPT task performance and mind wandering incidences was calculated using the number of new items added to the DPT activity list after the SART task was performed. As for experiments 1 and 2, the correlation was also performed using the non-parametric rank-correlation test, Kendall’s tau.

### The sustained attention to response task (SART)

The second phase of experiments 1, 2 and 3 was the sustained attention to response task (SART; Fig. 1a). This task required participants to continuously monitor a stream of stimuli presented at the center of a computer screen. Each stimulus was presented until the response for the maximum duration of two seconds (in case no response was provided). The inter-stimulus interval (ISI) was 2 seconds. For experiments 2 and 3 we used a classical digit SART task, whereas for experiment 1 we implemented a variant using word stimuli. These stimuli consisted of both target items (presented at a constant rate of 11%) and frequent non-target items (Fig. 1b). Participants were instructed to press the space bar whenever a non-target item was presented and withhold the button-press response when a target item was displayed. In experiment 1, the stimuli presented were contingent on the word item triplets from the CRA task. Participants were presented with individual words from the triplets that remained unsolved after participants had performed the CRA for the first time. These words were used as non-target items (i.e., frequent stimuli) along with nonsense words created using random strings of letters that served as target items. Participants were instructed to respond using the space bar when a semantically meaningful word was presented and withhold the button press whenever a nonsense word (random string) was shown. Only words used in CRA as puzzle words (and not the solution word) were presented. We hypothesized that by using words contingent to CRA items, creative insights may be triggered and would therefore aid in the participants’ ability to solve the compound word puzzles presented after the SART. The nonsense target words were generated by randomly shuffling letters from the triplet words, and keeping the number of letters in each word similar across both conditions. Participants were instructed to respond using the button press as quickly and as accurately as possible.

In experiments 2 and 3, participants were presented with a stream of digits (0–9) with the same constant target rate of 11%. Digits from 1 to 9 were used as non-target items (i.e., where participants were required to respond by pressing the space bar) and 0 was used as a target which required participants to withhold the button-press.

### The experience sampling procedure

During the SART task participants were examined for the contents of their experience. In order to investigate this, questions relating to the content of the participants’ mind wandering/ task-unrelated thoughts (TUTs) were presented at random intervals during the SART task, similar to other studies examining the effects of mind wandering (comprehensively reviewed in ref. 29). In our study, experience sampling probes were presented with an inter-probe interval, randomly distributed between 40 and 60 sec. We used 100 probes in experiments 1 and 2 and 40 probes in experiment 3. When the probes were presented, they prompted the participants to answer whether they were focused on the task or not immediately before the probe appeared (two alternative forced choice; experiments 1 and 2). In experiment 3 we used different probes to gain more information about the participant’s experience. The first probe posed a similar question as in experiments 1 and 2 (“Where was your attention focused immediately before the probe appeared”?). The participant was prompted with a choice of five possible answers to this question (five alternative forced choice responses): “I was focused on the task of pressing the correct button”; “I was focused on inner seeing”; “I was focused on inner feelings”; “I was focused on inner speech”; “I was focused on something else”. The participant could then freely choose whichever response was most appropriate. Subsequently, a second probe was displayed and participants were then required to select one out of the four following possibilities: “I was focused on the task”; “I was focused on the daily routine list”; “I was focused on the experiment”; “I was focused on something else”. Responses were recoded off-line into two categories, namely an *on the task* and *off the task* category. The *on task category* included the items “I was focused on the experiment”, “I was focused on the task” and “I was focused on the task of pressing the correct button”. The *off the task* category (mind wandering) included the items “I was focused on the daily routine list”, and “I was focused on something else”. For the correlation analysis we examined the number of times participants responded as being focused on anything else rather than being “focused on the task”. We used this extended probing procedure for two main reasons: (1) to acquire more information about the content of mind wandering (probe 1) and, (2) to refine the index used for the correlation analysis (probe 2). This exploratory probing method follows from our observation that participants are often confused as to the state of actually being *focused on the task* (i.e. on pressing the correct button) with a state of thinking *about the task* at hand (e.g. thinking about how dull the current task is; see also ref. 30). Participants were instructed to take their time when responding to the experience sampling probes in all three experiments.

### Analysis of reaction times

Reaction times for responses to the SART stimuli preceding the experience sampling probe over longer time intervals (i.e. the last five responses prior to the experience sampling probe; for similar approach see ref. 31) were considered for analysis. This interval is referred to as “time before the probe”. Comparisons of reaction times were calculated using Wilcoxon signed rank tests (on-task vs. off-task). Kruskal-Wallis tests were used to quantify whether there were any difference across time points before the mind wandering probe (responses to stimuli 1–5). We decided to use these non-parametric tests rather than analysis of variances because the assumptions of the ANOVA in terms of normality and of homogeneity of variances were violated. This was indicated by Kolmogorov-Smirnov and Levene’s tests.

We also compared the standard deviations of reaction times across the five consecutive responses preceding off- (mind wandering) and on-task (no mind wandering) state. To this end, we first averaged RTs across trials for each of the five responses and then calculated the standard deviation across these five reaction times. Finally, we used paired Wilcoxon signed rank to compare the standard deviation of reaction times between on- and off-task states. To calculate the effect sizes in each comparison which involved the median and the Wilcoxon signed rank test, we divided the value of the z-statistic by the square root of the total number of comparisons^32^: r = z/sqrt(n1 + n2).

### Data availability statement

The datasets analyzed during the current study are available from the corresponding author on reasonable request.
